# Unlocking the potential of targeting histone-modifying enzymes for treating IBD and CRC

**DOI:** 10.1186/s13148-023-01562-1

**Published:** 2023-09-11

**Authors:** Bing Liang, Yanhong Wang, Jiazhen Xu, Yingchun Shao, Dongming Xing

**Affiliations:** 1 Cancer Institute, The Affiliated Hospital of Qingdao University, Qingdao, China; 2 Qingdao Cancer Institute, Qingdao University, Qingdao, China; 3 School of Life Sciences, Tsinghua University, Beijing, China

**Keywords:** Histone modification, Histone-modifying enzyme, Gut microbiota, IBD, CRC

## Abstract

Dysregulation of histone modifications has been implicated in the pathogenesis of both inflammatory bowel disease (IBD) and colorectal cancer (CRC). These diseases are characterized by chronic inflammation, and alterations in histone modifications have been linked to their development and progression. Furthermore, the gut microbiota plays a crucial role in regulating immune responses and maintaining gut homeostasis, and it has been shown to exert effects on histone modifications and gene expression in host cells. Recent advances in our understanding of the roles of histone-modifying enzymes and their associated chromatin modifications in IBD and CRC have provided new insights into potential therapeutic interventions. In particular, inhibitors of histone-modifying enzymes have been explored in clinical trials as a possible therapeutic approach for these diseases. This review aims to explore these potential therapeutic interventions and analyze previous and ongoing clinical trials that examined the use of histone-modifying enzyme inhibitors for the treatment of IBD and CRC. This paper will contribute to the current body of knowledge by exploring the latest advances in the field and discussing the limitations of existing approaches. By providing a comprehensive analysis of the potential benefits of targeting histone-modifying enzymes for the treatment of IBD and CRC, this review will help to inform future research in this area and highlight the significance of understanding the functions of histone-modifying enzymes and their associated chromatin modifications in gastrointestinal disorders for the development of potential therapeutic interventions.

## Introduction

Epigenetics is the study of heritable changes in gene function without changes in DNA sequence [1]. Histone modifications, DNA modifications, and histone variants are commonly considered to be epigenetic changes that dynamically regulate the structure and function of chromatin in cells. Posttranslational modifications (PTMs) of histones, especially at their N-terminal tails, play important roles in regulating chromatin dynamics and a variety of DNA-templated biological processes. Dysregulation of these processes due to histone modifications is closely associated with the development of various diseases [2, 3]. At least nine different types of histone modifications have been discovered. Acetylation, methylation, phosphorylation, ubiquitylation, and SUMOylation are the most well understood modifications, while GlcNAcylation, citrullination, crotonylation, and isomerization are more recent discoveries that have yet to be thoroughly investigated. Each of these modifications is added or removed from histone amino acid residues by a specific set of enzymes. The chromatin-modifying enzymes that catalyze major PTMs involve histone acetyltransferases (HATs), histone deacetylases (HDACs), histone methyltransferases (HMTs), and histone lysine transferases (KATs). These enzymes can be recruited to target sites by sequence-specific DNA-binding transcription factors or more general features of the DNA, such as its global CG content and DNA methylation status, which can be read by the DNA-binding Zn-finger CxxC domain present in many chromatin-modifying enzymes [4–7]. Conversely, factors associated with the transcriptional machinery can directly lead to the accumulation of specific marks such as trimethylation of histone H3 lysine 4 (H3K4me3) and trimethylation of histone H3 lysine 36 (H3K36me3). Transcriptionally active and silent chromatin is characterized by distinct PTMs or combinations thereof [8].

Gastrointestinal disorders are prevalent and affect a significant portion of the global population, leading to substantial morbidity and mortality. Inflammatory bowel disease (IBD) affects over 3 million people in the USA alone and is a major cause of chronic disability [9]. Colorectal cancer (CRC) is the second leading cause of cancer deaths worldwide and is projected to cause over 1.5 million deaths annually by 2030 [10, 11]. These statistics underscore the need for a better understanding of the molecular mechanisms underlying these diseases and the development of potential therapeutic interventions. Although IBD and CRC are two different diseases, IBD patients have an increased risk of developing CRC [12]. The main mechanisms underlying IBD-CRC pathogenesis include inflammation and genetic susceptibility [12]. Recent studies have shown that dysregulation of histone modifications is closely associated with the pathogenesis of gastrointestinal disorders. Aberrant histone modifications have been identified in the colonic mucosa of patients with IBD and are thought to contribute to the chronic inflammation characteristic of these diseases [13, 14]. Similarly, alterations in histone modifications have been linked to the development and progression of CRC [15, 16]. These findings suggest that targeting histone-modifying enzymes and their associated chromatin modifications could be a promising therapeutic strategy for the treatment of gastrointestinal disorders.

Moreover, the development of host-microbe relationships between mammalian hosts and their beneficial symbiotic microorganisms has emerged as a critical factor in regulating inflammation in gastrointestinal disorders [17]. The gut microbiota, which includes bacteria, viruses, fungi, and other microorganisms, plays a key role in modulating immune responses and maintaining gut homeostasis [18]. Dysbiosis, or an imbalance in the gut microbiota, has been implicated in the pathogenesis of several gastrointestinal disorders, including IBD and CRC [19, 20]. Recent studies have shown that the gut microbiota can affect histone modifications and gene expression in host cells, highlighting the need to better understand the molecular mechanisms underlying these processes [21–23]. In this review, we explore recent advances regarding the roles of histone-modifying enzymes and their associated chromatin modifications in the pathogenesis of IBD and CRC. Our aim is to provide insights that could pave the way for the development of potential therapeutic interventions. Moreover, we conduct a critical analysis of previous and ongoing clinical trials that examined histone-modifying enzyme inhibitors for the treatment of IBD and CRC. We also highlight current challenges. Furthermore, we explore potential future directions and areas of research in this field. Ultimately, this review underscores the significance of comprehending the functions of histone-modifying enzymes and their associated chromatin modifications in gastrointestinal disorders for the development of potential therapeutic interventions.

## Histone-modifying enzymes represent a promising new avenue for the treatment of IBD

IBD is a complex chronic disorder characterized by inflammation of the gastrointestinal tract, including Crohn's disease (CD) and ulcerative colitis (UC). Although the precise pathogenesis of IBD remains unknown, it is believed to be a multifactorial disease involving the interplay between genetic, environmental, gut microbiota, and immune factors. Conventional treatments control IBD symptoms through pharmacotherapy, including aminosalicylates, corticosteroids, immunomodulators, and biologics [24]. However, despite the emergence of numerous therapeutic options for IBD over the past few years, several clinical questions have emerged regarding the optimal definitions of treatment success and the appropriate time to declare a treatment failure [25].

Epigenetic mechanisms, such as DNA methylation and noncoding RNAs, play a crucial role in the pathogenesis of IBD by causing changes in T-cell activity, cytokine production, and intestinal epithelial integrity, leading to chronic inflammation [13]. Recent studies have also highlighted the critical role of histone modifications in the pathogenesis of IBD [26]. Several histone modifications have been identified as key regulators of inflammatory responses and implicated in the pathogenesis of IBD [14]. These mechanisms provide a framework for understanding the pathogenesis and progression of IBD, including variations in the timing of disease onset [27]. HDACs and HMTs are two major classes of histone-modifying enzymes that are implicated in the regulation of gene expression associated with inflammation [14, 28–32]. While current research has made significant progress in understanding the genetic and environmental factors contributing to the development of IBD, the challenges of identifying novel therapeutic targets and developing effective treatments remain [33]. Therefore, targeting epigenetic regulatory factors, such as histone-modifying enzymes, represent a promising approach for the treatment of IBD. Modulating the expression and activity of these enzymes may enable the regulation of the expression of genes that play critical roles in inflammation and the immune response, thereby reducing the severity of IBD and improving patient outcomes.

### Microbial epigenetic regulation as a potential therapy for IBD

Recent research has uncovered the presence of specific epigenetic molecules and regulatory mechanisms influenced by the microbiota, which have shown significant potential in the treatment of IBD (Table 1). Multiple studies have demonstrated that the gut microbiota has a profound impact on the host's epigenetic landscape and immune function, thus providing new insights into potential therapies for IBD. For example, Kelly et al. [34] highlighted the potential for commensal microbes to modulate transcriptional output through epigenetics, revealing an H3K4me3 signature in the intestinal epithelial cells of IBD patients who can be regulated by microbiota. These findings may reveal how microbiota predispose individuals to subsequent intestinal inflammation and disease. Similarly, Lund et al. [35] showed that the gut microbiota can modulate the H4 acetylation pattern of colonic histones by microbiota-derived butyrate, and Yang et al. [36] discovered a mechanism by which butyrate produced by gut microbiota metabolism can inhibit HDAC and activate G-protein coupled receptor 41 (GPR41) to promote the expression of *hypoxia-inducible factor 1α (HIF1α)* and *aromatic hydrocarbon receptor (AhR)*. This, in turn, leads to HIF1α binding to the hypoxia response element (HRE) region of the interleukin-22 (IL-22) promoter and induces histone acetylation, enhancing *IL-22* expression and alleviating colitis in mice. IL-22 is a critical cytokine that promotes epithelial cell regeneration and mucosal barrier integrity in the intestines by activating signal transducer and activator of transcription 3 (STAT3) and stimulating the expression of antibacterial peptides and mucins [37], and it is highly upregulated in IBD patients [38]. Moreover, microbiota-derived inositol-1,4,5-trisphosphate (InsP3) through the metabolism of phytate antagonized the inhibitory effect of butyrate on intestinal histone deacetylase 3 (HDAC3) and induced HDAC3 activation, thereby promoting intestinal epithelial cell proliferation and intestinal damage repair, and may be used to improve IBD [39]. HDAC3 is essential for maintaining intestinal homeostasis and host defense, and the loss of HDAC3 can lead to inflammation and intestinal damage [40]. Gao et al. [41] reported that butyrate can reverse the increased levels of HDAC3 and phosphorylated-P65 (p-P65) proteins, which can prevent IBD by restoring intestinal homeostasis via the HDAC3-phosphorylated-glycogen synthase kinase 3 beta (p-GSK-3β)-β-catenin-Nuclear factor erythroid 2-related factor 2 (Nrf2)- Nuclear factor kappa-light-chain-enhancer of activated B cells (NF-κB) pathway. Moreover, Li et al. [42] discovered that the pan-HDAC inhibitor trichostatin A (TSA) can mimic the effects of butyrate. Additionally, propionate, another microbiota-derived short-chain fatty acid (SCFA), has been found to inhibit HDAC and suppress IL-17 production by intestinal γδ T cells from patients with IBD [43]. The IL-17 family, consisting of six members (IL-17A-IL-17F), plays a role in host defense against infections by inducing cytokines, chemokines, and antimicrobial proteins [44, 45]. *IL-17* expression is elevated in the mucosa of IBD patients [46]. Gut microbiota-derived acetate activated the *Drosophila* immunodeficiency (IMD) pathway in enteroendocrine cells (EECs) and induced chromatin remodeling within these cells through a Tip60 (also known as lysine acetyltransferase 5 [KAT5])-steroid hormone axis, which coregulates host metabolism and intestinal innate immunity in the anterior midgut [47]. Finally, Deng et al. [48] revealed that the probiotic protein p40 produced by *Lactobacillus rhamnosus* GG promotes Treg differentiation and alleviates colitis by enhancing mono- and trimethylation of histone H3 lysine 4 (H3K4me1/3) modification of histones on transforming growth factor beta (TGFβ) through increased expression of *Setd1β* (a methyltransferase in the COMPASS complex). Collectively, these findings suggest that targeting specific epigenetic mechanisms through microbial metabolites and regulatory molecules can be a promising therapeutic approach for treating IBD.

**Table 1:** Microbial metabolites-mediated epigenetic regulation in IBD

Study	Microbial metabolites	Histone modifying enzymes/histone markers	Regulatory molecules	Effects on IBD
Kelly et al. [34]	N/A	H3K4me3	N/A	Correlate with intestinal inflammation
Lund et al. [35]	Butyrate	H4 acetylation	N/A	Affect energy homeostasis
Yang et al. [36]	Butyrate	HDAC	GPR41-HIF1α/AhR-IL-22	Maintain intestinal homeostasis
Wu et al. [39]	InsP3	HDAC3	N/A	Promote intestinal epithelial cell proliferation and repair
Gao et al. [41]	Butyrate	HDAC3	p-GSK-3β, β-catenin, Nrf2, NF-κB	Reverse small intestinal mucosal injury, inflammation response, and oxidative stress
Li et al. [42]	Butyrate	HDAC	N/A	Ameliorate mucosal inflammation
Dupraz et al. [43]	Propionate	HDAC	IL-17	Inhibit intestinal inflammation
Jugder et al. [47]	Acetate	Tip60	IMD signaling	Control host metabolism and innate immunity in the gut
Deng et al. [48]	p40	Setd1β-mediated H3K4me1/3	TGFβ	Protect the gut against inflammation

### Epigenetic regulation and modulation of IBD through histone-modifying enzymes

**Fig. 1:**
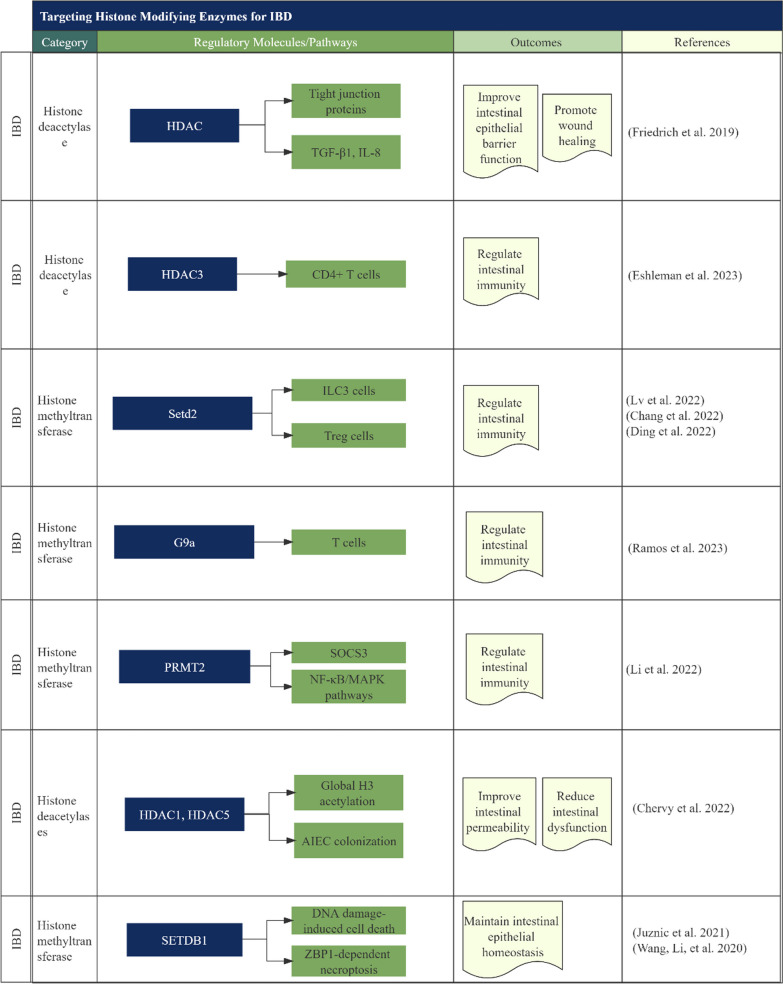
A schematic diagram showing the targeting of different histone-modifying enzymes and regulatory mechanisms for the treatment of IBD

Studies have investigated the role of different epigenetic molecules and regulatory mechanisms in the development and treatment of IBD and associated conditions (Fig. 1). Friedrich et al. [49] found that in colonic epithelial cells of patients with IBD, HDAC expression is downregulated, but HDAC inhibitors can improve the function of the intestinal epithelial barrier by modulating the expression of tight junction proteins (claudin-1, claudin-2, and occludin), TGF-β1 and IL-8 and ultimately promoting wound healing. IL-8, a neutrophil chemoattractant, is found in increased quantities in the inflamed mucosa of IBD patients [50, 51]. SET domain containing 2 (Setd2), which is a mammalian H3K36 methyltransferase, is critical in regulating epigenetic mechanisms affecting histone and DNA modifications that lead to the differentiation and function of group 3 innate lymphoid cell (ILC3) subpopulations (NKp46 [natural killer cell marker] + ILC3, double negative [DN] ILC3 and CCR6 [chemokine receptor] + ILC3), which are phenotypically and functionally heterogeneous in transcription factor expression, cytokine secretion, and spatial localization and play important regulatory roles in diseases such as intestinal infections, inflammatory diseases, and tumors [52]. A recently published work showed that Setd2 deficiency resulted in a decrease in intestinal ILC3 cells, with an increased proportion of NKp46 + ILC3 subpopulations and high expression of toxicity-related molecules (granzyme A and C). Additionally, Setd2 deletion decreased the number and function of CCR6 + ILC3 cells and affected the formation of intestinal isolated lymphoid tissue (SILT). Thus, Setd2 regulates gene expression by affecting the chromatin accessibility of ILC3 subpopulations, which provides insights into tumor immunotherapy and the pathogenesis of intestinal inflammation [53]. Furthermore, Setd2 was found to regulate intestinal Treg cells, which are important for their survival and suppression of colitis [54]. Setd2 promotes the expression of GATA binding protein 3 (GATA3) (transcription factor driving type 2 immunity) and suppression of tumorigenicity 2 (ST2) (receptor of IL-33), thus helping to transcribe target genes and regulate enhancer activity. These phenomena provide new insights into Treg cell regulation and intestinal immunity. T cells play an important role in the development of IBD, and chronic intestinal inflammation in IBD patients is associated with dysfunctional numbers and functions of effector and regulatory T cells residing in intestinal tissues. A recent study published in “Clinical Investigation” found that epithelial HDAC3 regulates the dynamic balance of CD4 + T-cell subsets, which allows them to recognize commensal bacteria and control inflammation [55]. Mice deficient in HDAC3 resulted in an increased accumulation of commensal-specific CD4 + T cells in their intestines, which in turn led to a decrease in commensal-specific regulatory T cells (Tregs) and an increase in T helper 17 cells (Th17), promoting T-cell-mediated colitis. Mechanistically, HDAC3 limits the number of commensal-specific T cells in the intestine by inducing major histocompatibility complex class II (MHC II) in epithelial cells through gut microbiota [55]. Previously, it was shown that histone methyltransferase G9a (also known as euchromatic histone-lysine N-methyltransferase 2 [EHMT2]) was associated with T-cell differentiation and fate decisions [56, 57]. A study in “Gastroenterology” found that regulating G9a in T cells affects their differentiation and can alleviate T-cell-associated colitis in mice. This is achieved by reducing dimethylation of histone H3 lysine 9 (H3K9me2) modification in T cells, activating the sterol regulatory element-binding protein (SREBP)-dependent cholesterol synthesis pathway, promoting differentiation to Treg cells, and alleviating 2,4,6-trinitrobenzene sulfonic acid (TNBS)-induced acute colitis. The study supports using G9a inhibitors to treat immune-related diseases such as IBD [58]. Sun et al. found that protein arginine methyltransferase 2 (PRMT2) adds the repressive histone mark asymmetric dimethylation of histone H3 arginine 8 (H3R8me2a) at the promoter region of the suppressor of cytokine signaling 3 promoter (SOCS3), thereby inhibiting the expression of SOCS3 and its downstream genes. The inhibition of *SOCS3* expression and the prevention of SOCS3 from degrading TNF receptor-associated factor 5 (TRAF5) through ubiquitination caused an increase in *TRAF5* expression. This, in turn, led to the activation of the downstream NF-κB/MAPK pathway through TRAF5 mediation. Overexpression of *PRMT2* exacerbates dextran sulfate sodium (DSS)-induced colitis, and conversely, knockdown of PRMT2 alleviates DSS-induced colitis. Therefore, targeting PRMT2 may be a potential strategy for the treatment of colitis [32]. Adherent invasive *Escherichia coli* (AIEC) colonizes abnormally in the ileal mucosa of patients with CD [59, 60]. The results of a study published in “Gut Microbes” found that acetylation levels of histone H3 were significantly upregulated in CD patients with AIEC colonization [61]. This study revealed that *HDAC1* expression is crucial in preventing AIEC from colonizing the intestinal mucosa. On the other hand, *HDAC5* expression promotes the invasion of AIEC. In CD patients, the expression of *HDAC1* and *HDAC5* was negatively and positively correlated with *Enterobacteria* load in ileal mucosa, respectively. Based on their observations, two potential approaches to limit the colonization of *Enterobacteria*—specifically, AIEC bacteria – could be to either enhance the expression or activity of HDAC1 in intestinal epithelial cells or to reduce the activity of HDAC5. SWI/SNF-related, matrix-associated actin-dependent regulator of chromatin, subfamily A, containing DEAD/H box 1 (Smarcad1), is a conserved chromatin remodeling factor that is highly expressed in the dry and proliferative regions of the intestinal epithelium, but its function in intestinal epithelial tissue is unknown. Kazakevych et al. discovered that intestinal epithelial cell-specific knockdown of Smarcad1 resulted in significant changes in chromosome accessibility and histone H3K9me3, regulated gene expression, upregulated genes related to natural immunity and inflammation, and alleviated microbiota-induced inflammation and colitis disease in mice [62]. Therefore, Smarcad1 and related microbiota may serve as pharmacological and probiotic therapeutic targets for inflammatory diseases. In addition, SET domain bifurcated histone lysine methyltransferase 1 (SETDB1) is a histone H3K9 methyltransferase that plays a regulatory role in intestinal epithelial homeostasis and IBD. From a study published in “Gut”, SETDB1 can maintain intestinal epithelial homeostasis by silencing endogenous retroviruses to inhibit DNA damage [63]. Mice specifically deficient in SETDB1 in the intestinal epithelium exhibited impaired intestinal epithelial differentiation, an impaired intestinal barrier, enhanced intestinal inflammation, and reduced survival. Additionally, a number of missense mutations associated with SETDB1 deficiency in function were significantly enriched in IBD patients. Taken together, the results of this study suggest that SETDB1 may be a potential target for the treatment of IBD [63]. Furthermore, another study found that deletion of SETDB1 leads to genomic instability of intestinal stem cells and release of endogenous retrovirus, which triggers Z-DNA-binding protein 1 (ZBP1)-dependent necroptosis and intestinal inflammation; this potential pathogenic mechanism of IBD provides an important new idea for the treatment of IBD [64].

## Histone-modifying enzymes as potential therapeutic targets for CRC

**Fig. 2:**
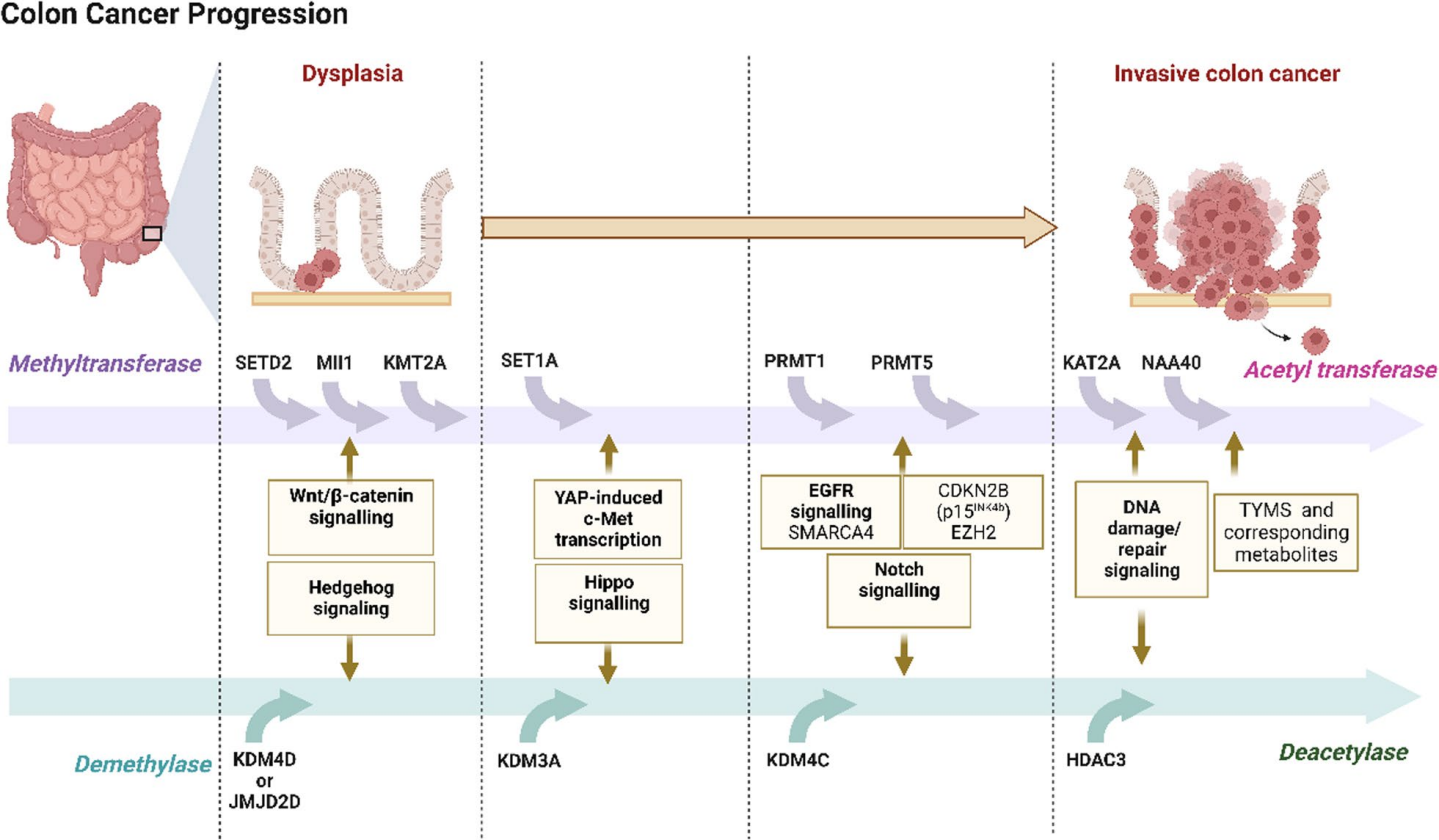
A schematic diagram showing different histone-modifying enzymes involved in the progression of CRC indicated by dysplasia (left) and invasive colon cancer (right). Histone-modifying enzymes are categorized into 4 groups: methyltransferase, demethylase, acetyltransferase, and deacetylase. From the left to the right, different histone-modifying enzymes regulate different signaling pathways leading to CRC progression but are not associated with the progression process. This figure is adapted from “Colon Cancer Progression” by BioRender.com (2023). Retrieved from https://app.biorender.com/biorender-templates

The ideal treatment for CRC is to achieve complete tumor and metastasis removal through surgical intervention, which is often necessary [65]. Epigenetic modifications, including histone modifications, play a crucial role in CRC progression (Fig. 2). In CRC patients, the H3K36 methyltransferase Setd2 is expressed at low levels, and in vitro experiments indicate that Setd2 deficiency promotes cell growth and migration. Researchers have demonstrated that Setd2 deletion accelerates colon carcinogenesis and progression through conditional knockout and spontaneous colon cancer mouse models, thus highlighting the critical role of Setd2 in CRC development [66]. The underlying mechanism is that Setd2 affects the shearing of H3K36 regulatory genes, which reduces DVL2 pre-mRNA intron retention, ultimately promoting the Wnt/β-catenin signaling pathway and tumorigenesis. Moreover, the involvement of lysine demethylase 4D (KDM4D or JMJD2D) in Wnt/β-catenin signaling has been shown to demethylate H3K9me2/3 and promote gene expression, indicating its essential role in CRC cell lines [67, 68]. Further studies have found that TNFα induces histone demethylase *JMJD2D* expression in colitis, and inhibition of *JMJD2D* expression suppresses Hedgehog signaling, leading to the inhibition of CRC growth and metastasis [69]. Additionally, lysine demethylase 3A (KDM3A), a mono- and dimethylation of histone H3 lysine 9 (H3K9me1/2) demethylase, facilitates *Yes-associated protein 1 (YAP1)* expression, thus enhancing H3K27 acetylation modification in hippo target gene enhancers; furthermore, KDM3A promotes hippo target gene expression, which may facilitate the development of CRC [70]. Another recent study revealed that N-alpha-acetyltransferase 40 (NAA40) affects global histone methylation and CRC cell survival through its acetyltransferase activity in stimulating transcription of the one-carbon metabolic gene thymidylate synthase (TYMS), whose product is targeted by 5-fluorouracil (5-FU) [71]. Similarly, AT-rich interaction domain-containing protein 3B (ARID3B) is crucial for the growth of CRC and promotes the stem-like features of CRC by activating Notch target genes, intestinal stem cell (ISC) genes, and programmed death-ligand 1 (PD-L1) through the recruitment of lysine-specific demethylase 4C (KDM4C) to modulate the chromatin configuration for transcriptional activation [72]. Furthermore, the tumor suppressor abhydrolase domain containing 5 (ABHD5) interacts with DPY30, the core subunit of the SET domain containing 1A (SET1A) methyltransferase complex, promoting its ubiquitination and degradation in the cytoplasm, thereby inhibiting SET1A and upregulating c-Met expression, which promotes stemness in CRC cancer cells [73]. Another critical protein, protein arginine methyltransferase 1 (PRMT1), has been shown to mediate asymmetric dimethylation of histone H4 arginine 3 (H4R3me2a), which recruits SWI/SNF-related, matrix-associated, actin-dependent regulator of chromatin, subfamily A, member 4 (Smarca4), an ATPase subunit in the chromatin remodeling complex SWI/SNF, to enhance epidermal growth factor receptor (EGFR) signaling, thereby promoting the proliferation and migration of CRC cells [74]. Interestingly, the downregulation of PRMT1 has also been found to induce CRC cell apoptosis [75]. Furthermore, protein arginine methyltransferase 5 (PRMT5), a type II arginine methyltransferase, has been identified as a promoter of CRC cell proliferation through its interaction with enhancer of zeste homolog 2 (EZH2) to inhibit *cyclin-dependent kinase 4 inhibitor B (CDKN2B)* expression [76]. Hence, targeting PRMT1/Smarca4 and PRMT5/EZH2 may represent promising strategies for CRC treatment. Another important histone methyltransferase, lysine-specific methyltransferase 2A (KMT2A), which is responsible for the methylation of histone H3 (H3K4me), is highly expressed in leucine-rich repeat-containing G-protein coupled receptor 5 (Lgr5) + stem cells and human colon cancer and is associated with poorer survival in colon cancer patients [77]. KMT2A regulates the expression of *Gata4/6*, which promotes Wnt-driven intestinal tumorigenesis, by converting polycomb repressive complex 2 (PRC2)-mediated repressive trimethylation of histone H3 lysine 27 (H3K27me3) to activating H3K4me3. Furthermore, KMT2A has been found to promote Wnt/β-catenin-mediated transcription of target genes by facilitating β-catenin to occupy the active promoter of H3K4me3 modification. Targeting KMT2A may therefore selectively inhibit the growth of β-catenin-dependent CRC [78]. Notably, high expressions of PRMT5, EZH2, and KMT2A in CRC patients have been found to be correlated with a worse prognosis [76]. Finally, recent investigations into the potential of sulforaphane (SFN) as a modifier of HDAC and HAT activity have yielded promising results. P300/CBP-associated factor (PCAF) (also known as lysine acetyltransferase 2B [KAT2B]) and lysine acetyltransferase 2A (KAT2A/GCN5) are closely related proteins that belong to an evolutionarily conserved family of histone acetyltransferases with important roles in transcriptional activation, cell cycle arrest, and cell differentiation [79, 80]. PCAF has been found to be epigenetically downregulated in CRC and to increase the resistance of CRC to 5-FU [81]. KAT2A has been found to be overexpressed in CRC and affects tumor metabolic reprogramming in CRC progression through epigenetic activation of E2F transcription factor 1 (E2F1) [82]. SFN analogs have been found to alter HAT/HDAC activities, histone acetylation status, and associated DNA damage/repair signaling pathways, leading to a reduction in the expression of HDAC3, PCAF, and KAT2A in CRC cells. These findings suggest that modulation of HAT/HDAC activities could represent a viable therapeutic strategy for CRC [83].

## Clinical trials investigating histone-modifying enzyme inhibitors for IBD and CRC

Unlike genetic mutations, epigenetic alterations are reversible, making them an attractive target for therapeutic intervention. Histone-modifying enzymes, which play a crucial role in regulating gene expression and chromatin structure, have emerged as promising druggable targets, as evidenced by the approval of eight agents in three epigenetic target classes, including HDAC, DNMT, and EZH2 inhibitors, for the treatment of different malignancies by the US FDA. Among the five approved HDAC inhibitors, vorinostat (Zolinza), also known as SAHA, has been the most successful and was approved for cutaneous T-cell lymphoma in 2006. Other approved agents, such as belinostat (Beleodaq), panobinostat (Farydak), tucidinostat (Epidaza), and romidepsin (Istodax), with varying specificity and routes of administration, have been approved for different indications, including hematological neoplasms such as T-cell lymphomas and multiple myeloma [84]. Moreover, azacytidine (Vidaza) and decitabine (Dacogen), two DNMT inhibitors, are approved for the treatment of myelodysplastic syndrome [85], while tazemetostat (Tazverik), an EZH2 inhibitor, has been approved for the treatment of metastatic or locally advanced epithelioid sarcoma [86].

In recent years, there has been a surge of interest in exploring epigenetic therapies as potential treatment options for IBD and CRC. The rationale for targeting histone-modifying enzymes stems from their central role in epigenetic regulation. Histone acetylation and deacetylation—which are facilitated by HATs and HDACs, respectively – modulate chromatin accessibility and gene expression. Dysregulation of these processes has been implicated in the pathogenesis of both IBD and CRC [16, 87, 88]. Aberrant histone modifications can lead to altered expression of proinflammatory cytokines and oncogenes, thus promoting inflammation and tumor growth [14, 89]. By selectively inhibiting HDACs and other histone-modifying enzymes, it is possible to restore normal gene expression patterns, thereby attenuating inflammation and halting cancer progression.

### Clinical trials in IBD treatment

Among the clinical trials exploring histone-modifying enzyme inhibitors for IBD treatment, the clinical trial NCT00792740 [90] evaluated the effect of ITF2357 on mucosal healing in patients with moderate-to-severe active CD. ITF2357, an orally active synthetic inhibitor of HDAC enzymes, has shown promise in selectively inhibiting proinflammatory cytokine production in vitro and displaying anti-inflammatory effects in both animal models and humans. The study was conducted in up to 25 clinical sites in Europe, with patients randomly assigned to receive either ITF2357 or a placebo for 8 consecutive weeks followed by a 4-week follow-up. Although the trial was terminated due to futility based on an interim analysis of the first 40 patients, it provided crucial insights into the potential of histone-modifying enzyme inhibitors in IBD treatment, laying the groundwork for future investigations. Additionally, the ongoing trial NCT03167437 [90], an open-label, proof-of-consent study conducted by the National Institute of Allergy and Infectious Diseases (NIAID), aimed to assess vorinostat, a synthetic HDAC inhibitor, for the treatment of moderate-to-severe CD and maintenance therapy with ustekinumab. The results of these trials will aid in understanding the efficacy and safety of targeting histone-modifying enzymes in IBD treatment.

### Clinical trials in CRC treatment

In the realm of CRC treatment, the clinical trial NCT01105377 [91], conducted by the National Cancer Institute (NCI), explored the combination of azacitidine and entinostat in patients with metastatic CRC. The primary objective was to determine the preliminary efficacy of this combination by assessing time to progression and toxicity as secondary endpoints. Although the trial did not demonstrate sufficient activity in unselected patients, it provided valuable data for refining future treatment strategies. Moving forward, researchers undertook NCT03215264 [92] to evaluate regorafenib, hydroxychloroquine (HCQ), and entinostat in metastatic CRC. HCQ, a disease-modifying anti-rheumatic drug (DMARD), and regorafenib, a kinase inhibitor, were used in combination with entinostat. The trial's findings, published in "The Oncologist," revealed that the combination was well tolerated and showed preliminary evidence of clinical activity in patients with metastatic CRC, offering promise for potential future treatment options [93]. Moreover, the ongoing trial NCT05694936 [94], a phase II randomized controlled trial led by the Australasian Gastrointestinal Trials Group, explored the combination of the HDAC inhibitor sodium valproate (VPA) with anti-EGFR monoclonal antibody (panitumumab or cetuximab) maintenance in the first-line treatment of patients with RAS wild-type metastatic CRC. The trial's primary objective was to evaluate progression-free survival (PFS); the secondary endpoints were overall survival (OS), objective response rates (ORRs), and safety. In parallel, NCT02437136 [95], a phase 1b/2 dose-escalation study conducted by Syndax Pharmaceuticals, investigated the combination of entinostat with pembrolizumab in non-small cell lung cancer (NSCLC) and expansion cohorts in NSCLC, melanoma, and CRC. The trial aimed to determine the safety and tolerability of this combination and assess its effectiveness in patients with NSCLC, melanoma, and mismatch repair-proficient CRC. Additionally, the EMERGE trial (NCT03812796) [96], conducted by the Royal Marsden NHS Foundation Trust, evaluated the efficacy of domatinostat, a selective HDAC1 inhibitor, in combination with avelumab, a PD-L1 antibody, in patients with microsatellite stable CRC or esophagogastric adenocarcinoma (OGA) previously treated with chemotherapy. The trial's results, published in ESMO Congress 2021, deemed the combination to be safe and led to phase IIb cohort expansion for OGA and CRC [97]. The results of these trials will shed light on the potential of histone-modifying enzyme inhibitors as novel therapeutic approaches for CRC.

### Challenges in developing and conducting clinical trials for histone-modifying enzyme inhibitors

Histone modifications play a pivotal role in the maintenance of gut health; however, their precise influence remains elusive, thus posing a challenge to the development of targeted therapies for IBD and CRC. Additionally, the gut microbiota is known to modulate histone modifications, but the underlying mechanisms have yet to be fully elucidated, thus underscoring the importance of a comprehensive understanding of their interplay in order to develop effective therapeutic interventions. Compounding these challenges is the intricacy of histone modifications themselves, which can occur at multiple sites on the histone protein and have opposing effects on gene expression [98]. Thus, the development of inhibitors targeting specific modifications while avoiding interference with others is a formidable task.

Further complexity arises from the diversity of histone-modifying enzymes, each with unique substrate specificity and mechanisms of action, making it difficult to target individual enzymes without disrupting other enzymes and pathways [99]. Additionally, the potential off-target effects, variable metabolism, and optimal dosing and treatment duration of histone-modifying enzyme inhibitors are challenging to address [99, 100, 101]. Consequently, designing clinical trials for these inhibitors entails complexities, including patient selection, biomarker identification, and careful monitoring of potential side effects and long-term effects [102, 103]. As histone-modifying enzyme inhibitors continue to be developed and evaluated, careful attention must be paid to the intricate interplay between histone modifications, gut microbiota, and patient outcomes to ensure their safe and effective use.

## Future directions

In the context of this review on the potential of histone-modifying enzyme inhibitors for the treatment of IBD and CRC, it is imperative to contemplate the potential future directions in this field. One key direction for future research is the development of more specific and targeted inhibitors that can avoid off-target effects. Maximizing efficacy while minimizing the risk of unintended harm to patients is critical. Another promising area of research is the identification of biomarkers that can better assess treatment response. Accurate measurement of treatment response could inform the development of personalized medicine approaches that take into account individual variability in response to these inhibitors. Additionally, combination therapies targeting multiple histone-modifying enzymes or pathways could be a promising avenue for future research. By combining targeted therapies, we can achieve greater efficacy while minimizing the risk of unintended harm. Further research is needed to better understand the role of the gut microbiome in influencing treatment outcomes. This could include exploring the potential for microbiome-based therapies that work in conjunction with histone-modifying enzyme inhibitors to enhance treatment efficacy.

In conclusion, we firmly believe that the potential of histone-modifying enzyme inhibitors for the treatment of IBD and CRC is an exciting area of research with significant promise. While there are certainly challenges that need to be addressed, we remain optimistic about the potential impact that these inhibitors could have on improving outcomes for patients with these conditions. The development of more specific and targeted inhibitors, the identification of biomarkers, and combination therapies are all avenues for continued research in this field. Ultimately, we must continue to invest in this research to unlock the full potential of histone-modifying enzyme inhibitors and improve the lives of those living with IBD and CRC.

## Data Availability

All data generated or analyzed during this study are included in this published article.

## Abbreviations

TNBS: 2,4,6-Trinitrobenzene sulfonic acid
5-FU: 5-Fluorouracil
ABHD5: Abhydrolase domain containing 5
AIEC: Adherent invasive *Escherichia coli*
AhR: Aromatic hydrocarbon receptor
H3R8me2a: Asymmetric dimethylation of histone H3 arginine 8
H4R3me2a: Asymmetric dimethylation of histone H4 arginine 3
ARID3B: AT-rich interaction domain-containing protein 3B
CRC: Colorectal cancer
CD: Crohn’s disease
CDKN2B: Cyclin-dependent kinase 4 inhibitor B
SOCS3: Cytokine signaling 3 promoter
DSS: Dextran sulfate sodium
H3K9me2: Dimethylation of histone H3 lysine 9
DMARD: Disease-modifying anti-rheumatic drug
E2F1: E2F transcription factor 1
EECs: Enteroendocrine cells
EGFR: Epidermal growth factor receptor
GATA3: GATA binding protein 3
GPR41: G-protein coupled receptor 41
ILC3: Group 3 innate lymphoid cells
HATs: Histone acetyltransferases
HDACs: Histone deacetylases
KATs: Histone lysine transferases
HMTs: Histone methyltransferases
HCQ: Hydroxychloroquine
HRE: Hypoxia response element
HIF1α: Hypoxia-inducible factor 1α
IMD: Immunodeficiency
IBD: Inflammatory bowel disease
InsP3: Inositol-1,4,5-trisphosphate
IL-22: Interlukin-22
SILT: Intestinal isolated lymphoid tissue
ISC): Intestinal stem cell
Lgr5: Leucine-rich repeat-containing G-protein coupled receptor 5
H3K4: Lysine 4 on histone H3
KAT2A: Lysine acetyltransferase 2A
KAT2B: Lysine acetyltransferase 2B
KAT5: Lysine acetyltransferase 5
KDM3A: Lysine demethylase 3A
KDM4D or JMJD2D: Lysine demethylase 4D
KDM4C: Lysine-specific demethylase 4C
KMT2A: Lysine-specific methyltransferase 2A
MHC II: Major histocompatibility complex class II
Mll1: Mixed-lineage leukemia 1
H3K4me1/3: Mono and trimethylation of histone H3 lysine 4
NAA40: N-alpha-acetyltransferase 40
NCI: National Cancer Institute
NIAID: National Institute of Allergy and Infectious Diseases
NSCLC: Non-small cell lung cancer
Nrf2: Nuclear factor erythroid 2-related factor 2
NF-κB: Nuclear factor kappa-light-chain-enhancer of activated B cells
ORRs: Objective response rates
OGA: Oesophagogastric adenocarcinoma
OS: Overall survival
PCAF: P300/CBP-associated factor
p-GSK-3β: Phosphorylated-glycogen synthase kinase 3 beta
p-P65: Phosphorylated-P65
PRC2: Polycomb repressive complex 2
PTMs: Post-translational modifications
PD-L1: Programmed death-ligand 1
PFS: Progression-free survival
PRMT1: Protein arginine methyltransferase 1
PRMT2: Protein arginine methyltransferase 2
PRMT5: Protein arginine methyltransferase 5
RP2D: Recommended phase II dose
Tregs: Regulatory T cells
SETDB1: SET domain bifurcated histone lysine methyltransferase 1
SET1A: SET domain containing 1A
Setd2: SET domain containing 2
SCFA: Short-chain fatty acid
STAT3: Signal transducer and activator of transcription 3
VPA: Sodium valproate
SREBP: Sterol regulatory element-binding protein
SFN: Sulforaphane
ST2: Suppression of tumorigenicity 2
Smarca4: SWI/SNF related, matrix associated, actin dependent regulator of chromatin, subfamily A, member 4
Smarcad1: SWI/SNF-related, matrix-associated actin-dependent regulator of chromatin, subfamily A, containing DEAD/H box 1
Th17: T helper 17 cells
TRAF5: TNF receptor-associated factor 5
TYMS: Transcription of the one-carbon metabolic gene thymidylate synthase
TGFβ: Transforming growth factor beta
TSA: Trichostatin A
H3K27me3: Trimethylation of histone H3 lysine 27
H3K36me3: Trimethylation of histone H3 lysine 36
H3K4me3: Trimethylation of histone H3 lysine 4
UC: Ulcerative colitis
YAP1: Yes-associated protein 1
ZBP1: Z-DNA-binding protein 1
EZH2: Zeste homolog 2
